# Corticosteroids in childhood epilepsies: A systematic review

**DOI:** 10.3389/fneur.2023.1142253

**Published:** 2023-03-10

**Authors:** Lena-Luise Becker, Angela M. Kaindl

**Affiliations:** ^1^Department of Pediatric Neurology, Charité – Universitätsmedizin Berlin, Berlin, Germany; ^2^Center for Chronically Sick Children, Charité – Universitätsmedizin Berlin, Berlin, Germany; ^3^German Epilepsy Center for Children and Adolescents, Charité – Universitätsmedizin Berlin, Berlin, Germany; ^4^Institute of Cell- and Neurobiology, Charité – Universitätsmedizin Berlin, Berlin, Germany

**Keywords:** corticosteroid, seizure, epileptic spasm, DEE-SWAS, drug-resistant epilepsy (DRE), pediatric, epilepsy

## Abstract

Corticosteroids have been used for the treatment of patients with epilepsy for more than 6 decades, based on the hypothesis of inflammation in the genesis and/or promotion of epilepsy. We, therefore, aimed to provide a systematic overview of the use of corticosteroid regimes in childhood epilepsies in line with the PRISMA guidelines. We performed a structured literature search *via* PubMed and identified 160 papers with only three randomized controlled trials excluding the substantial trials on epileptic spasms. Corticosteroid regimes, duration of treatment (days to several months), and dosage protocols were highly variable in these studies. Evidence supports the use of steroids in epileptic spasms; however, there is only limited evidence for a positive effect for other epilepsy syndromes, e.g., epileptic encephalopathy with spike-and-wave activity in sleep [(D)EE-SWAS] or drug-resistant epilepsies (DREs). In (D)EE-SWAS (nine studies, 126 patients), 64% of patients showed an improvement either in the EEG or in their language/cognition following various steroid treatment regimes. In DRE (15 studies, 436 patients), a positive effect with a seizure reduction in 50% of pediatric and adult patients and seizure freedom in 15% was identified; however, no recommendation can be drawn due to the heterozygous cohort. This review highlights the immense need for controlled studies using steroids, especially in DRE, to offer patients new treatment options.

## Introduction

Epilepsy affects ~41–187 in 1,00,000 children, with the highest incidence in the first year of life (1). Particularly in drug-resistant epilepsies (DREs), the number of patients with epilepsy syndromes and encephalopathies not responding to anti-seizure medication (ASM) has not changed within the last 30 years, despite new drugs with multiple targets being available on the market (2). When the first ASM fails to achieve seizure freedom, the second and third ASMs have a likelihood of seizure freedom of 11%−13% and 3%−4%, respectively (2, 3).

**Figure 1:**
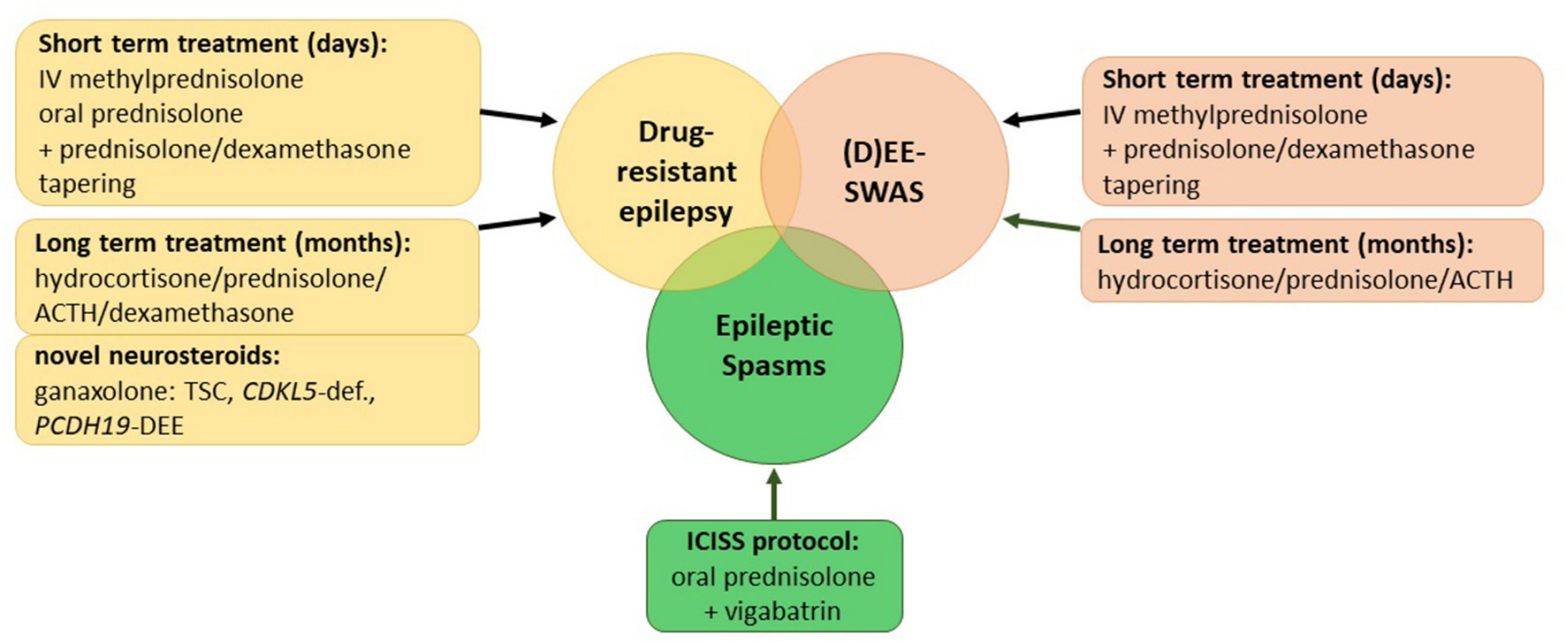
Overview of current corticosteroid regimes in childhood epilepsies. (D)EE-SWAS, developmental and epileptic encephalopathy with spike-and-wave activation in sleep; TSC, tuberous sclerosis complex.

Corticosteroids have been used for the treatment of patients with epilepsy for over 60 years. In 1958, Sorel and Dusaucy-Bauloye first reported a marked improvement in 21 patients with epileptic spasms treated with ACTH (4). Since then, corticosteroids have evolved into an essential component of the standard therapy of epileptic spasms backed up by evidence of randomized controlled trials (RCTs) (5, 6). Nevertheless, for other childhood epilepsies, various treatment algorithms exist that encompass several steroids including prednisolone, adrenocorticotrophin hormone (ACTH), methylprednisolone, hydrocortisone, and the novel neurosteroid ganaxolone as well as a multifold of protocols on their application (Supplementary Table 1). This review aims to summarize the current literature on steroid treatments in childhood epilepsies (Figure 1).

### Inflammation in epilepsy

The use of steroids is based on findings on inflammatory processes in epileptogenesis (7). There is evidence that seizures are associated with inflammation and *vice versa* (8). Particularly in prolonged seizures, e.g., in status epilepticus and drug-resistant epilepsy, the release and upregulation of pro-inflammmatory cytokines [IL-1β, TNF, IL-6, prostaglandin E2, and a high-mobility group box 1 (HMGB1) complement system] have been studied not only in rodents but also in the brain samples of patients (upregulation of IL-1β, HMGB1, IL-1R1, TLR4) and in the CSF of humans (high IL-6) (8–11). In rodents, this inflammation affects seizure reoccurrence and severity, and it has been hypothesized that this model is also present in human epilepsy syndromes (8–10). One of the most severe epilepsy syndromes, Rasmussen encephalitis, is a long-known example of this mechanism leading to DRE with focal-onset seizures and status epilepticus (epilepsia partialis continua), hemiparesis, and cognitive regression (12). Immunosuppression and hemispherotomy are the therapeutic approaches for this disease that is largely of unknown cause (12). Moreover, antibody-mediated epilepsies such as anti-NMDA receptor encephalitis or anti-AMPA receptor encephalitis often lead to DRE and status epilepticus (13, 14). The removal of the autoantibody will usually cure the DRE in these cases (14).

Although the exact mechanism of how steroids modulate seizure frequency is unknown, several hypotheses exist (7). By interaction with the γ-aminobutyric acid (GABA)_A_ receptor, steroids prolong the duration and frequency of the ligand-gated chloride channel opening, thereby suppressing a possible hyperexcitability (15).

### Common side effects

Corticosteroids are usually well-tolerated; however, moderate to severe adverse effects (AEs) can occur, and patients should be educated. Known AEs associated with short-term treatment include hyperglycemia and electrolyte imbalance, acute infections including pneumonia and sepsis, impaired wound healing, psychological effects, and behavioral changes such as agitation, irritability, and psychosis (16, 17). In addition, AEs with long-term treatment include cushingoid features such as weight gain, growth retardation, glycosuria/hyperglycemia, type 2 diabetes, hypertension, and bone fractures/osteoporosis. Furthermore, long-term treatment can lead to a suppression of the hypothalamus and pituitary gland, leading to adrenal insufficiency, an increased risk of infection, psychosis, nephrocalcinosis, cataract, and brain atrophy (18–20).

## Methods

We searched PubMed with the MeSH terms “epilepsy” and “steroids” and “child” or “infant” or “adolescence” or “child, preschool” between 1 January 2000 and 13 September 2022 for randomized controlled trials (RCTs), clinical trials, observational studies, case series with more than or equal to two patients receiving any corticosteroid treatment (prednisolone, hydrocortisone, dexamethasone, methylprednisolone, ACTH, and ganaxolone) for the treatment of childhood epilepsy. Studies were excluded if only one patient was reported, another treatment regime than the aforementioned regime was administered, and the publication language was not English. Information collected included the number of patients, the study type (retrospective, single/multicenter, randomized controlled study, etc.), type of epilepsy/epilepsy syndrome, treatment regime including the dosage and duration of treatment, observation period, and outcome. The outcome was reported heterogeneously, without a homogenous follow-up. We, therefore, extracted the number of reported patients with either seizure freedom or reduction (seizure reduction ranging from 50 to 80%) and/or an improvement of cognition/EEG. The reoccurrence of seizures after a period of seizure freedom was extracted, if possible, from the studies. Because structured meta-analysis for epileptic spasms exists, no analysis was performed on this cohort (5, 6). The PRISMA flowchart is shown in Supplementary Figure 1. Included studies were divided into subgroups for infantile epileptic spasms syndrome (IESS), epilepsy syndromes with spike-and-wave activity in sleep (SWAS), Lennox–Gastaut syndrome (LGS), Angelman syndrome, and other DRE types. In the subgroup of DRE epilepsies, we differentiated them into two groups: a group receiving short-term treatments over 3–10 days (IV methylprednisolone and prednisolone p.o.) and a group that was administered with long-term treatments over several months. The statistical analysis in these groups was limited to descriptive analyses performed with Microsoft^®^ Excel for Mac (version 16.68). Apart from studies on IESS, only three RCTs were identified that employed different steroids (ACTH, hydrocortisone, deflazacort, and ganaxolone) in two different epilepsy subgroups, namely, status epilepticus, DRE, and CDKL5-deficiency disorder (21–23). In the latter, no statistical analysis was performed due to the non-comparability of the studies. The review was not registered, and no protocol was prepared.

## Results

### Infantile epileptic spasms syndrome

**Figure 2:**
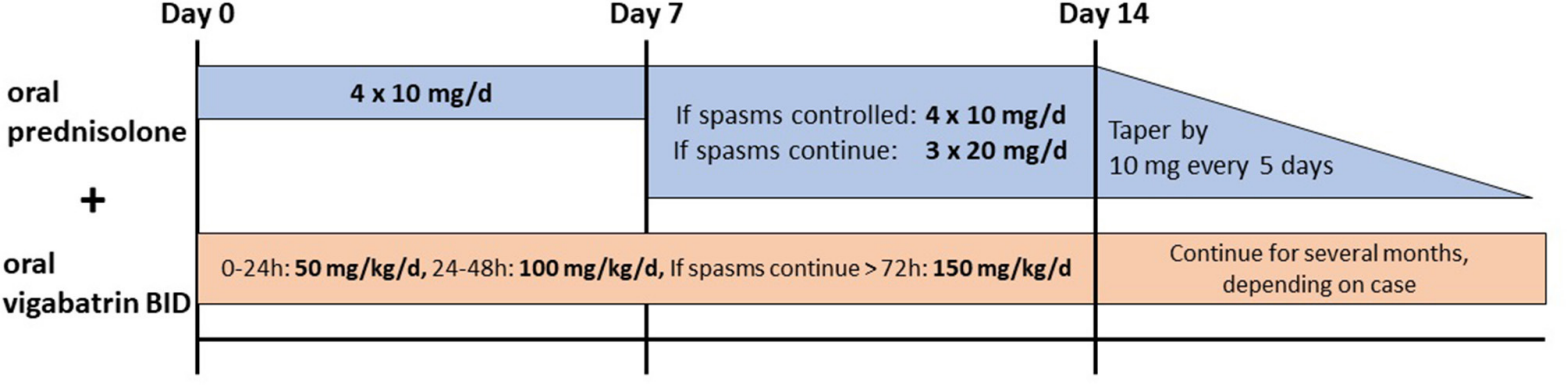
Recommended treatment for epileptic spasms by the International Collaborative Infantile Spasms Study (ICISS) (26).

Infants with IESS develop epileptic spasms usually within the first year of life with or without hypsarrhythmia in the interictal EEG and various degrees of developmental delay (24). Corticosteroids or ACTH are broadly used in the treatment of epileptic spasms; however, still, various protocols and different steroids are used. A recent meta-analysis by Guang et al. revealed that prednisolone, independent of the dose, was as effective as ACTH in controlling spasms (6, 25–28). Hormonal treatments are also superior to vigabatrin-only treatment, as shown in many studies (6). A combination therapy of vigabatrin and hormonal therapy is more efficient in controlling spasms than ACTH (6). A commonly used protocol is that of the International Collaborative Infantile Spasms Study (ICISS, Figure 2) (26). Oral prednisolone is given (10 mg, four times/day) for 2 weeks, and if spasms do not cease on day 7 or reappear, the dosage should be increased to 20 mg, three times/day. In addition, oral vigabatrin is started with 50 mg/kg/day and increased to 150 mg/kg/day in two doses/day within 4 days if spasms do not cease on day 7 or reappear (26). After 2 weeks, the hormonal treatment is tapered off by 10 mg every 5 days, and vigabatrin treatment is continued (26). There is evidence that in the long term, there is no difference in seizure frequency and cognitive development on comparing the combination of vigabatrin and hormonal therapy and vigabatrin alone (29, 30).

### Epilepsy syndromes with spike-and-wave activity in sleep

Developmental and epileptic encephalopathy (DEE) with spike-and-wave activity in sleep (SWAS, formally status epilepticus in sleep = ESES; DEE-SWAS) and epileptic encephalopathy with SWAS (EE-SWAS) is a severe and mostly treatment-resistant epilepsy syndrome with an overall developmental regression (31). In the EE-SWAS subtype of Landau–Kleffner syndrome (LKS), the regression affects predominantly language (31). Although currently, no controlled trials exist on corticosteroid treatment in these entities, steroids are used in many centers for SWAS and LKS (32). Studies with long-term treatment over several months show promising effects of different steroid regimes hydrocortisone (33), ACTH (34), prednisolone, e.g., 1 mg/kg/day (35), intravenous (IV) methylprednisolone 20 mg/kg/day for 3 days with a gradual withdrawal with oral prednisolone (36), oral dexamethasone for 0.15 mg/kg/day (37) (Supplementary Table 1). The largest study by Chen et al. showed an improvement in 22 of 27 patients with ESES (SWAS) and six of six patients with LKS when treated with oral prednisolone at 1–2 mg/kg/day for 6 months (38). Buzatu et al. (33) (hydrocortisone 5 mg/kg/day for 1 month followed by tapering off for 9 months) found 44 patients with SWAS with an improvement in 21 patients but relapse in 14 of 21 patients.

In a total of 126 patients from nine studies with SWAS or LKS, 81 patients (64%) showed an improvement either in the EEG or their language/cognition following various steroid treatment regimes. In 17 patients (56%) of two publications of the nine studies, a relapse within the observation period was described (33–42). In a recent pooled analysis of 950 treatments including standard ASM, benzodiazepines, and steroids in 575 cases with (D)EE-SWAS reported in 112 articles, a superior effect of steroids (*n* = 166) in comparison to standard ASM and benzodiazepines was revealed (43). However, the authors highlight caution due to the small, retrospective heterogeneous cohort that also included single case reports (32). Currently, a still ongoing multicenter randomized controlled trial comparing corticosteroids (monthly IV methylprednisolone pulse or daily oral prednisolone) vs. clobazam aims to give new perspectives for the treatment of ESES (RESCUE ESES) (32). This ongoing trial is greatly needed to support the heterogeneous literature on steroid treatment in patients with SWAS.

### Lennox–Gastaut syndrome

Lennox–Gastaut syndrome is a DEE with a childhood onset characterized by multiple drug-resistance seizure semiologies including tonic seizures, cognitive impairment, and diffuse slow spike-and-wave and generalized fast activity on EEG (31). The differentiation of LGS vs. other severe epilepsies is crucial for correct treatment (31). Only two retrospective studies were identified. Either oral prednisolone (2 mg/kg/day for 6 weeks, followed by tapering off) or ACTH (0.25–0.75 mg/day for 10–57 days) was given. In 41 of 77 patients (53%), seizure freedom directly following the treatment was obtained; however, in only 12 of 77 patients, the seizure freedom persisted after a variable observation period between 9 months and 7 years (44, 45).

### Angelman syndrome

Only one case series of four patients with 2 mg/kg/day oral prednisolone describes generally an initial good response on alertness and seizure frequency in all patients. However, in 75% of patients, the seizures reoccurred after weaning off. Therefore, in two patients, the authors describe that the patients received long-term treatment with 1–2 mg/kg/day oral prednisolone on alternating days, and this resulted in seizure freedom (46).

### Drug-resistant epilepsies

If two correctly chosen and dosed ASM fail to achieve seizure freedom, the epilepsy is referred to as DRE. Despite many new second-generation ASMs, the proportion of DRE has not decreased within the last 30 years with only 11.6 and 4.4% likelihood of seizure freedom if the second or third ASM fails (2, 47). There are multiple very heterogeneous retrospective studies describing the effect of steroids not only in pediatric cohorts but also in mixed pediatric and adult cohorts (*n* = 15, total 436 patients), and only one doubleblind crossover study from Pentella et al. “exists” (21) (ACTH i.m. 5 mg for 2 weeks and 10 mg for 2 weeks vs. placebo). Corticosteroids used in the included studies ranged from hydrocortisone (5–20 mg/kg/day), prednisolone (1–2 mg/kg/day), methylprednisolone (20–30 mg/kg/day), dexamethasone (0–5 mg/kg/day), and ACTH (25–40 U/day) either orally, intramuscular, or intravenously ranging from a treatment duration from 3 days to 1 year (Supplementary Table 1). Such heterogeneous treatment protocols even within one study and further literature highlight the lack of a consensus on steroid treatment even within one hospital, despite the broad use of DRE. Steroid treatment-associated seizure reduction of ~50%−80% was reported in 219 of 436 patients (50%) and seizure freedom in 67 of 436 patients (15%) after variable observation periods between 3 months and 8 years. Only three studies informed about a reoccurrence of seizures in 17 of 25 patients (68%) (48–50). Furthermore, no obvious difference was found between the short and long-term treatment duration subgroup: (i) short-term treatment duration subgroup with three studies included (51–53) seizure reduction in 34 of 77 patients (44%) and seizure freedom in seven of 77 patients (9%) and (ii) long-term treatment duration subgroup with 12 studies (23, 38, 48–50, 54–59) included seizure reduction in 185 of 359 patients (52%) and seizure freedom in 60 of 359 patients (17%). However, due to the heterogeneity of these studies, no recommendations with respect to better treatment duration and regimen can be drawn.

### Status epilepticus

In the initial phase of a status epilepticus (SE), steroids are not part of existing treatment algorithms. If the SE continues under conventional ASM and a refractory SE (RSE > 120 min) or super-refractory SE (SRSE > 24 h) is diagnosed, steroids are listed as therapeutic options in many reviews (9, 60). New-onset refractory SE (NORSE) was newly defined as a clinical presentation without active epilepsy or other preexisting relevant neurological disorder with new-onset RSE without a clear cause (61, 62). Febrile infection-related epilepsy syndrome (FIRES) is a subcategory of NORSE in patients with a prior febrile infection, 2–24 weeks before the onset of SE (61).

Currently, evidence of treatment efficiency in all of these SE subtypes is based on published heterogeneous case series rather than randomized controlled trials. Therefore, there is no standardized protocol on steroid regime, dosage, and treatment duration (60, 62). Especially in the devastating life-threatening event of RSE/SRSE, controlled studies on steroid treatment are crucial to verify the efficiency and safety of this commonly used treatment.

### Novel neurosteroids

Ganaxolone, a member of the novel neuroactive steroids, interacts with synaptic and extrasynaptic γ-aminobutyric acid (GABA)_A_ receptors and various other ligands and other voltage-gated ion channels, thereby modulating specifically the GABA_A_ neuronal network. A first nonrandomized pilot study in 15 patients with refractory epilepsy showed a >50% reduction in seizure frequency in four patients (25%) (63). Different studies in SE randomized therapy in status epilepticus (RAISE) trial (NCT04391569), tuberous sclerosis complex (TSC), cyclin-dependent kinase-like 5 (*CDKL5*)-deficiency disorders, and protocadherin-19 (*PCDH19*)-related epilepsy are currently analyzing the effect of ganaxolone in these epilepsy subtypes (64).

CDKL5-deficiency disorder is an X-linked, DEE with a heterogeneous seizure semiology and mostly DRE (22). Ganaxolone (maximum dose of 63 mg/kg/day for patients weighing ≤ 28 kg or 1,800 mg/day for patients weighing >28 kg over 17 weeks) was recently tested in 101 patients with CDKL5*-*deficiency disorders in a randomized, placebo-controlled phase 3 trial (22). Ganaxolone was associated with a significantly better seizure frequency reduction than placebo (−30.7 vs. −6.9%). In an ongoing open-label extension study, long-term effects are addressed (22).

*PCDH19* variants cause early infantile epileptic encephalopathy 9 (EIEE9) with typical clusters of febrile and afebrile focal seizures (65). An open-label, uncontrolled phase 2 trial of a 26-week ganaxolone treatment resulted in a reduction of >50% in four of 11 patients. The double-blind, placebo-controlled phase 2 VIOLET study is completed, and the first data showed a seizure reduction of >50% in five of 10 patients in the ganaxolone cohort in comparison to four of 11 patients in the placebo group (NCT03865732).

In TSC, seizures occur partly due to a disruption of the GABAergic interneurons within tubera (66). The current first-line treatment vigabatrin acts on the disrupted mTOR pathway as well as on GABA receptors (64). Currently, the data of the open-label phase 2, add-on trial (NCT04285346) showed a seizure reduction of >50% in 30.4% of 22 patients (http://marinuspharma.com/wp-content/uploads/2021/12/83028-GNX-TSC-AES-Poster_Final_2021-11-05_FINAL.pdf). In an open-label, uncontrolled phase ½ study, brexanolone, a proprietary formulation of allopregnanolone, was assessed in SRSE in a mixed pediatric and adult cohort with promising effects: 17 of 22 patients responded to the add-on treatment and 16 patients (73%) could be weaned of anesthetic agents. In a double-blind, controlled phase 3 trial, brexanolone was tested against a placebo and did not result in a better weaning off anesthetic agents in the brexanolone group in comparison with the placebo group (43.9 vs. 42.4%, NCT02477618) (67).

## Conclusion

Some retrospective studies show good response rates of various steroid treatment regimens in various epilepsy syndromes (Figure 2). However, there is a lack of results from RCT providing clear evidence for the use of specific steroid regimens in epilepsies other than epileptic spasms. The limiting factors of this review were the very heterogenous, small study cohorts with a high variability of study types, follow-up periods, treatment regimes, outcome data, and completeness of reported data sets. Therefore, no recommendations or statistics including a meta-analysis could be performed. Additional placebo-controlled studies are, therefore, needed. Several studies are underway with promising putative results for the epilepsy community. One of these is the randomized, controlled RESUE ESES trial comparing clobazam to steroids. In the future, studies on the dosage and duration of treatment are needed to establish a treatment guideline.

## Author contributions

L-LB and AMK were involved in conceptualization, investigation, collection of data, validation, writing the original draft, and in the review and editing process. Both authors contributed to the article and approved the submitted version.

## Supplementary material

Supplementary Figure 1 PRISMA flowchart. Page MJ, McKenzie JE, Bossuyt PM, Boutron I, Hoffmann TC, Mulrow CD, et al. The PRISMA 2020 statement is an updated guideline for reporting systematic reviews. *BMJ*. (2021) 372:n71. doi: 10.1136/bmj.n71.

Supplementary Table 1 Included studies in this review. AD, on alternating days; CSWS, continuous spike-wave during sleep; d, day; DRE, drug-resistant epilepsy; EE, epileptic encephalopathy; i.m., intramuscular; y, years; LKS, Landau–Kleffner syndrome; m: month; RCT: randomized controlled trial; retro: retrospective. p.o.: per oral.
