# Supplementary figures and images for: Corticosteroids in childhood epilepsies: A systematic review
Source: Front Neurol. 2023 Mar 10;14:1142253. doi: 10.3389/fneur.2023.1142253 (PMC10036579; doi:10.3389/fneur.2023.1142253)

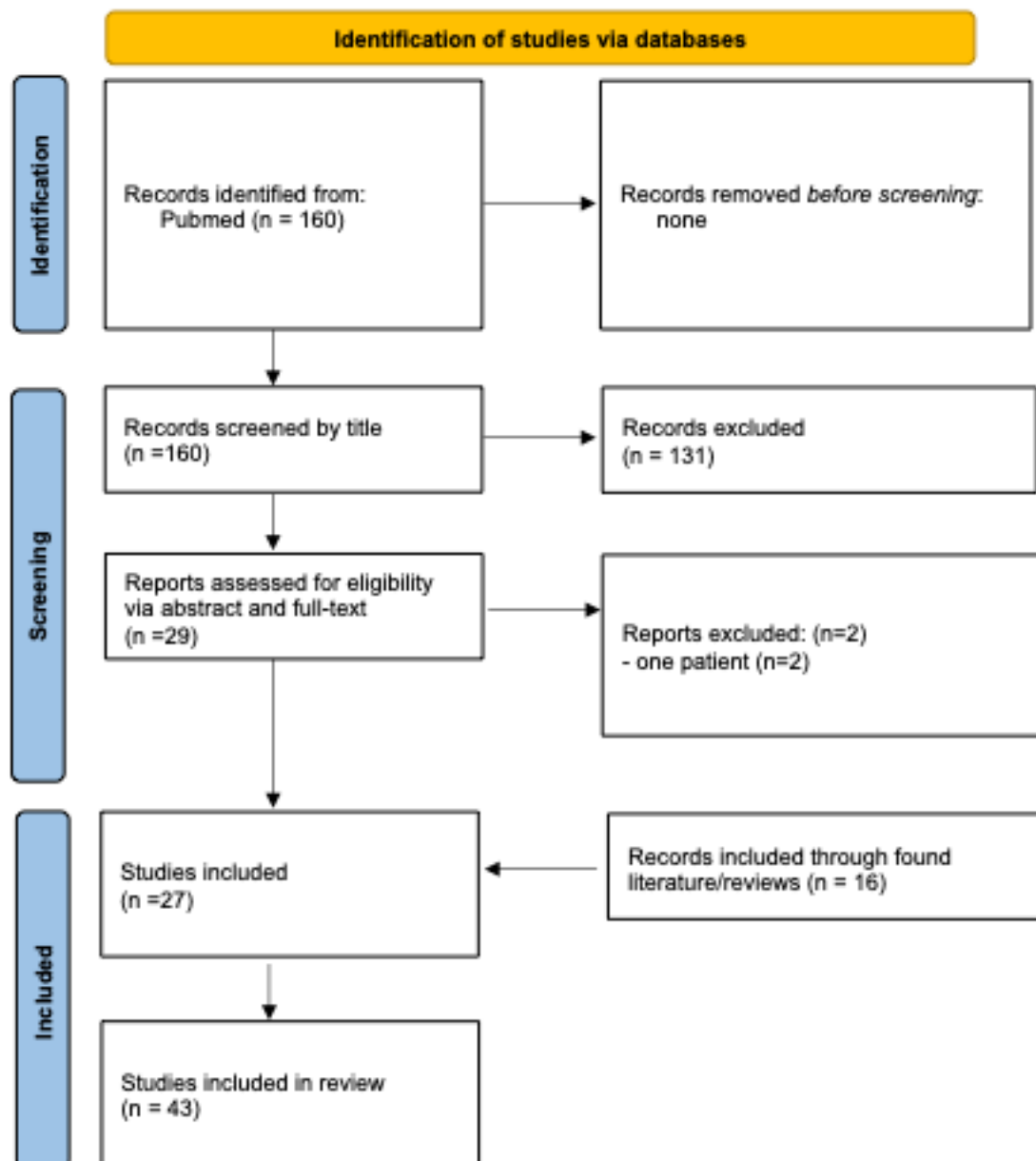

Supplement: Supplementary Figure 1 — PRISMA flowchart. Page MJ, McKenzie JE, Bossuyt PM, Boutron I, Hoffmann TC, Mulrow CD, et al. The PRISMA 2020 statement is an updated guideline for reporting systematic reviews. BMJ. (2021) 372:n71. doi: 10.1136/bmj.n71. [file Data_Sheet_1.PDF]
