# Supplementary material for: Corticosteroids in childhood epilepsies: A systematic review
Source: Front Neurol. 2023 Mar 10;14:1142253. doi: 10.3389/fneur.2023.1142253 (PMC10036579; doi:10.3389/fneur.2023.1142253)
Supplement: Supplementary Table 1 — Included studies in this review. AD, on alternating days; CSWS, continuous spike-wave during sleep; d, day; DRE, drug-resistant epilepsy; EE, epileptic encephalopathy; i.m., intramuscular; y, years; LKS, Landau–Kleffner syndrome; m: month; RCT: randomized controlled trial; retro: retrospective. p.o.: per oral. [file Table_1.docx]

**Supplemental Table 1: Included studies in this review.** AD, on alternating days; CSWS, continuous spike-wave during sleep. d: days; DRE, drug-resistant epilepsy; EE, epileptic encephalopathy; i.m.: intramuscular; y: years; LKS, Landau-Kleffner syndrome; m: months; RCT: randomized controlled trial; retro: retrospective. p.o.: per oral; SRSE: super refractory status epilepticus.

| **Study** | **Study**  **type** | **No. of pat.** | **Epilepsy classification** | **Treatment regime** | **Observational period** | **Outcome** | | |
| --- | --- | --- | --- | --- | --- | --- | --- | --- |
|  |  |  |  |  |  | **Seizure reduction 50-80 %** | **Seizure freedom** | **Seizure reoccurrence** |
| **Epileptic spasms** | | | | | | | | |
| Haberlandt et al. 2010^1^ | retro | 28 | Epileptic Spasms | i.m. ACTH 15-120 IU/d for 6 w followed by tapering off vs. i.v. 5 x dexamethasone 20 mg/m² for 3 d | missing |  | 9/11 vs. 4/7 |  |
| Al-Shehhi et al, 2021^2^  Wanigasinghe et al 2021^3^ | retro  retro,  follow-up | 65 | Epileptic Spasms refractory to vigabatrin | p.o. prednisolone  8 mg/kg/d for 2 w followed by tapering off | 6 w  4 y |  | 30  28 |  |
| Callaghan et al 2017  Callaghan et al 2018^4,5^ | open-label, RCT  follow-up | 377  362 | Epileptic Spasms | p.o. prednisolone 4x 10 mg/d 2w ( up to 3 x 20 mg) or i.m. tetracosactide depot 0·5 mg (40 IU) AD for 2 w vs. with vigabatrin  followed by tapering off | 4 w  18 m |  | 108/191 vs. 133/186  150/178 vs. 153/180 |  |
| Gowda et al, 2018^6^ | single center, randomized | 33 | Epileptic Spasms | i.m. ACTH 100 U/m² for 2 w vs. p.o. prednisolone 4 mg/kg/d for 2 w followed by tapering off | 6 m |  | 9/18 vs.  6/15 |  |
| Hodgeman et al 2016^7^ | retro | 57 | Epileptic Spasms | i.m. ACTH 150 U/m² for 2 w, 30 U/m² for 3 d, 15 U/m² for 3 d, 10 U/m² for 3 d, and 3x 10 U/m² AD | 14 d + 3 m |  | 40 | 8/40 |
| Gonzalez-Giraldo et al 2018^8^ | retro | 87 | Epileptic Spasms | p.o.prednisolone  40-60 mg/d for 2 w followed by tapering off | 3 m |  | 54 |  |
| Mohamed et al., 2011^9^ | retro | 75 | Epileptic Spasms | p.o. prednisolone 2-4 mg/kg/d or 4x 10 mg/d 2w vs. vigabatrin vs. valproate | 13 d |  | 11/18 vs. 23/54 vs. 2/3 | 4/11 vs. 7/23 vs. 1/2 |
| Lux et al., 2004^10^  Darke et al., 2010^11^ | RCT | 107  77 | Epileptic Spasms | vigabatrin (max 15 mg/kg/d) or prednisolone p.o. 4 x 10 mg/d (max. 3 x 20 mg) for 2w / Tetracosactide depot i.m. (0,5 mg [40 IU] AD for 1 w, 0·75 mg [60 IU] AD for 1 w followed by tapering off | 13 d  4 y |  | 28/52 vs. 40/55  67/77 |  |
| Chellamuthu et al., 2014^12^ | RCT, open label | 63 | Epileptic Spasms | prednisolone p.o. high-dose 4 mg/kg/d vs. low dose 2 mg/kg/d | 14 d | 11/31 vs. 8/32 | 16/31 vs. 8/32 |  |
| Kapoor et al., 2021^13^ | RCT, open label | 60 | Epileptic Spasms | methylprednisolone i.v. 30 mg/kg/d for 3 d vs. prednisolone p.o.4 mg/kg/d for 2 w followed by tapering off | 14 d |  | 17/31 vs. 20/29 | 6/31 vs. 0 after 6 w |
| **SWAS (n=132)** | | | | | | **Normalization EEG** | **Improvement of cognition/EEG** | **Re-occurrence of ESES/ seizures** |
| Buzatu et al., 2009 ^14^ | retro | 44 | CSWS | hydrocortisone 5 mg/kg/d for 1 months followed by tapering off for 9 months | 1 y | 21 |  | 14 |
| Lerman et al., 1991^15^ | retro | 4 | LKS | ACTH vs. high-dose prednisolone | 3-6 y |  | 4 |  |
| Sinclair et al, 2005^16^ | retro | 10 | CSWS/LKS | prednisolone p.o. 1 mg/kg/d for 6 m | 1-10 y |  | 9 |  |
| Robinson et al., 2001 ^17^ | retro | 18 | LKS | steroids | 12-180 m |  | 2 |  |
| Tsuru et al, 2000^18^ | retro | 2 | LKS | 3 x Methylprednisolone i.v. 20 mg/kg/d for 3 d and prednisolone p.o. 2 mg/kg/d for 1 m followed by tapering off | 2 m and 1 y |  | 2 |  |
| Fatema et al, 2015^19^ | retro | 3 | CSWS | Methylprednisolone i.v. for 3 d | missing | 1 |  |  |
| Chen et al., 2016^20^ | retro | 15 | CSWS | dexamethasone p.o. 0,15 mg(kg/d for 4 w | 6 m |  | 9 | 3 |
| Chen et al., 2014^21^ | retro | 33 | CSWS/LKS | prednisolone p.o. 1-2 mg/kg/d for 6 m | 1 m |  | 28 |  |
| Marescau et al., 1990 ^22^ | retro | 5 | LKS | steroids | missing |  | 3 |  |
| Haberlandt et al., 2010^1^ | retro | 5/28 | CSWS/LKS | ACTH i.m. 15-120 IU/d for 6 w followed by tapering off vs. i.v. 5 x dexamethasone 20 mg/m² for 3 d | missing |  | 2 |  |
| **Lennox-Gastaut Syndrome (n=77)** | | | | | **Observational period** | **Outcome** | | |
|  |  |  |  |  |  | **Seizure reduction 50-80 %** | **Seizure freedom** | **Seizure reoccurrence** |
| You et al., 2007 ^23^ | retro | 32 | LGS | Prednisolone 2 mg/kg/d for 6 w m followed by tapering off 2 w | 14-90 m | 23 | 19 | 7/19 |
| Yamatogi et al., 1979^24^ | retro | 45 | LGS | ACTH i.v. 0,25-0,75 mg | 9 m-7 y |  | 23 | 5/23 |
| **Angelman Syndrome** | | | | | | | | |
| Forrest et al, 2009^25^ | retro | 4 | Angelmann | prednisolone p.o. | missing | 4 |  |  |
| **Drug-resistant epilepsies** | | | | | | | | |
| **Short duration (3-10d, n=77)** | | | | | | | | |
| Pera et al., 2015^26^ | retro | 11 | EE | Methylprednisolone i.v. 15-30 mg/kg/d for 3d | 8-16 m | 8 | 4 |  |
| Kimizu et al 2020^27^ | retro | 31 | focal onset impaired awareness seizure | methylprednisolone i.v. 15-30 mg/kg/d for 3d |  | 10 | 3 |  |
| Gofshteyn et al., 2021^28^ | retro | 35 | DRE | dexamethasone p.o. 16 mg/d for 2 d, 12 mg/d for 2 d, 8 mg/d for 2 d, 5 mg/d fpr 2 d, 2 mg/d for 2 d, vs methylprednisolone i.v. 30 mg/kg/d for 3 d |  | 16 |  |  |
| **Long duration (n= 401)** | | | | | | | | |
| Hasaerts et al., 1989, ^29^ | retro | 32 | Sek. Gen. seizures | hydrocortisone p.o. 5 mg/kg/d for 1 m followed by tapering off 6 m | 7 m | 16 |  |  |
| Pentella et al.,1981^30^ | Double,  blind, crossover trial | 4 | DRE | ACTH i.m. 5 mg for 2 w and 10 mg for 2 w vs. placebo | 1 m |  |  |  |
| Sinclair et al, 2003^31^ | retro | 28 | DRE, absence | prednisolone p.o. 1 mg/kg/day for 12 w (6 w daily and 6 weeks AD) | 1-5 y | 11 | 12 |  |
| Verhelst et al., 2005^32^ | retro | 32 | DRE | n=13 dexamathasone 0.5—5 mg/kg/d from 3d to 8 m, n=10 hydrocortisone 5-20 mg/kg/d for 4w-20m, n=8 prednisolone 0,3-3 mg/kg/d) 7d-24 m, 1x methylprednisolone 2 mg/kg/d for 2 m, 4x  ACTH 2—5 IU/kg/d for 3 w | 6 m-8 y | 13 | 9 | 6/9 |
| Grosso et al, 2008^33^ | RCT, open, non-blinded | 35 | DRE | hydrocortisone p.o. 10 mg/kg for 1 m, 5 mg/kg/d y for 1 m, 2.5 mg/kg/d for 1  m, 1 mg/kg/d for 1 month, and 1 mg/kg AD 2 m  deflazacort: 0.75 mg/kg fpr 6 m | 12 m | 16 |  |  |
| Inui et al., 2015 ^34^ | retro | 2 | Lissencephaly, atypical absence seizures | 2 cycles ACTH i.m 0.015 mg/kg/d followed by a tapering off | 1 y |  | 2 |  |
| Kalra et al., 2009 ^35^ | retro | 2 | DRE | ACTH i.m. 40 IU/d for 4 wk followed by tapered off over next 8 wk | 4-6 m |  | 2 |  |
| Chatterjee et al., 2021 ^36^ | retro | 97 | EE | methylprednisolone i.v. 3d for 7.72 ± 6.25 months | 3 m | 64 | 24 |  |
| Charuvanij et al., 1992 ^37^ | retro | 21 | DRE | ACTH i.m.1.3-2.6 IU/kg for 5 w | 4 m-12 y | 5 |  | 4/5 |
| Bakker et al., 2015^38^ | retro | 26 | DRE | methylprednisolone i.v. 20 mg/kg/d followed by tapered off over next 12 w | 4 m | 9 | 6 |  |
| Nasiri et al., 2017 ^39^ | retro | 25 | DRE | methylprednisolone 25 m/kg/d for 1 w followed by tapered off | 3 m | 11 |  | 7/11 |
| Chen et al., 2014^21^ | retro | 49 | BECT | prednisolone 1-2 mg/kg/d for 6 m | 6 m | 40 |  |  |
| Gobbi et al., 2014^40^ | retro | 6 | epilepsy with frontal lobe seizures | ACTH i.m. 0.01 mg/kg/d for 2w, followed by tapering for 6 w | 12 m |  | 5 |  |
| **Other indications** | | | | |  | **Outcome** |  |  |
| Pieribone et al., 2007^41^ | Open-label | 15 | DRE | ganaxolone 12 mg/kg/d | 10 w | >50 % reduction: n=4 |  |  |
| Knight et al., 2022^42^ | RCT, placebo | 101 | CDKL5-deficiency disorder | ganaxolone p.o.max. 63 mg/kg/d ≤28 kg or 1800 mg/d f >28 kg over 17 w vs. placebo | 17 w | seizure frequency reduction: 30.7 % vs. -6.9 % |  |  |
| Rosenthal et al., 2017^43^ | Open-label | 25 | SRSE | Loading: brexanolone i.v. 286.6lg/kg for 1 hour, maintainance 4d: 86 or 156lg/kg/h | 29 d | weaning of TLA: n=17 |  |  |

**References:**

1. Haberlandt E, Weger C, Sigl SB, et al. Adrenocorticotropic hormone versus pulsatile dexamethasone in the treatment of infantile epilepsy syndromes. Pediatr Neurol. 2010;42(1):21-27.

2. Al-Shehhi W, Chau V, Boyd J, et al. Treatment with High-Dose Prednisolone in Vigabatrin-Refractory Infantile Spasms. Can J Neurol Sci. 2022;49(4):532-539.

3. Wanigasinghe J, Arambepola C, Ranganathan SS, Jayasundara K, Weerasinghe A, Wickramarachchi P. Epilepsy Outcome at Four Years in a Randomized Clinical Trial Comparing Oral Prednisolone and Intramuscular ACTH in West Syndrome. Pediatr Neurol. 2021;119:22-26.

4. O'Callaghan FJK, Edwards SW, Alber FD, et al. igabatrin with hormonal treatment versus hormonal treatment alone (ICISS) for infantile spasms: 18-month outcomes of an open-label, randomised controlled trial. Lancet Child Adolesc Health. 2018;2(10):715-725.

5. O'Callaghan FJ, Edwards SW, Alber FD, et al. Safety and effectiveness of hormonal treatment versus hormonal treatment with vigabatrin for infantile spasms (ICISS): a randomised, multicentre, open-label trial. Lancet Neurol. 2017;16(1):33-42.

6. Gowda VK, Narayanaswamy V, Shivappa SK, Benakappa N, Benakappa A. Corticotrophin-ACTH in Comparison to Prednisolone in West Syndrome - A Randomized Study. Indian J Pediatr. 2019;86(2):165-170.

7. Hodgeman RM, Kapur K, Paris A, et al. Effectiveness of once-daily high-dose ACTH for infantile spasms. Epilepsy Behav. 2016;59:4-8.

8. Gonzalez-Giraldo E, Stafstrom CE, Stanfield AC, Kossoff EH. Treating Infantile Spasms with High-Dose Oral Corticosteroids: A Retrospective Review of 87 Children. Pediatr Neurol. 2018;87:30-35.

9. Mohamed BP, Scott RC, Desai N, Gutta P, Patil S. Seizure outcome in infantile spasms--a retrospective study. Epilepsia. 2011;52(4):746-752.

10. Lux AL, Edwards SW, Hancock E, et al. The United Kingdom Infantile Spasms Study comparing vigabatrin with prednisolone or tetracosactide at 14 days: a multicentre, randomised controlled trial. Lancet. 2004;364(9447):1773-1778.

11. Darke K, Edwards SW, Hancock E, et al. Developmental and epilepsy outcomes at age 4 years in the UKISS trial comparing hormonal treatments to vigabatrin for infantile spasms: a multi-centre randomised trial. Arch Dis Child. 2010;95(5):382-386.

12. Chellamuthu P, Sharma S, Jain P, Kaushik JS, Seth A, Aneja S. High dose (4 mg/kg/day) versus usual dose (2 mg/kg/day) oral prednisolone for treatment of infantile spasms: an open-label, randomized controlled trial. Epilepsy Res. 2014;108(8):1378-1384.

13. Kapoor D, Sharma S, Garg D, et al. Intravenous Methylprednisolone Versus Oral Prednisolone for West Syndrome: A Randomized Open-Label Trial. Indian J Pediatr. 2021;88(8):778-784.

14. Buzatu M, Bulteau C, Altuzarra C, Dulac O, Van Bogaert P. Corticosteroids as treatment of epileptic syndromes with continuous spike-waves during slow-wave sleep. Epilepsia. 2009;50 Suppl 7:68-72.

15. Lerman P, Lerman-Sagie T, Kivity S. Effect of early corticosteroid therapy for Landau-Kleffner syndrome. Dev Med Child Neurol. 1991;33(3):257-260.

16. Sinclair DB, Snyder TJ. Corticosteroids for the treatment of Landau-kleffner syndrome and continuous spike-wave discharge during sleep. Pediatr Neurol. 2005;32(5):300-306.

17. Robinson RO, Baird G, Robinson G, Simonoff E. Landau-Kleffner syndrome: course and correlates with outcome. Dev Med Child Neurol. 2001;43(4):243-247.

18. Tsuru T, Mori M, Mizuguchi M, Momoi MY. Effects of high-dose intravenous corticosteroid therapy in Landau-Kleffner syndrome. Pediatr Neurol. 2000;22(2):145-147.

19. Fatema K, Rahman MM, Begum S. Characteristics and Management of Children with Continuous Spikes and Waves during Slow Sleep. Mymensingh Med J. 2015;24(4):806-812.

20. Chen J, Cai F, Jiang L, Hu Y, Feng C. A prospective study of dexamethasone therapy in refractory epileptic encephalopathy with continuous spike-and-wave during sleep. Epilepsy Behav. 2016;55:1-5.

21. Chen J, Yang Z, Liu X, et al. [Efficacy of methylprednisolone therapy for electrical status epilepticus during sleep in children]. Zhonghua Er Ke Za Zhi. 2014;52(9):678-682.

22. Marescaux C, Hirsch E, Finck S, et al. Landau-Kleffner syndrome: a pharmacologic study of five cases. Epilepsia. 1990;31(6):768-777.

23. You SJ, Jung DE, Kim HD, Lee HS, Kang HC. Efficacy and prognosis of a short course of prednisolone therapy for pediatric epilepsy. Eur J Paediatr Neurol. 2008;12(4):314-320.

24. Yamatogi Y, Ohtsuka Y, Ishida T, et al. Treatment of the Lennox syndrome with ACTH: a clinical and electroencephalographic study. Brain Dev. 1979;1(4):267-276.

25. Forrest KM, Young H, Dale RC, Gill DS. Benefit of corticosteroid therapy in Angelman syndrome. J Child Neurol. 2009;24(8):952-958.

26. Pera MC, Randazzo G, Masnada S, et al. Intravenous methylprednisolone pulse therapy for children with epileptic encephalopathy. Funct Neurol. 2015;30(3):173-179.

27. Kimizu T, Takahashi Y, Oboshi T, et al. Methylprednisolone pulse therapy in 31 patients with refractory epilepsy: A single-center retrospective analysis. Epilepsy Behav. 2020;109:107116.

28. Hasaerts D, Dulac O. [Hydrocortisone therapy of secondary generalized epilepsy in children]. Arch Fr Pediatr. 1989;46(9):635-639.

29. Pentella K, Bachman DS, Sandman CA. Trial of an ACTH4-9 Analogue (ORG 2766) in children with intractable seizures. Neuropediatrics. 1982;13(2):59-62.

30. Sinclair DB. Prednisone therapy in pediatric epilepsy. Pediatr Neurol. 2003;28(3):194-198.

31. Verhelst H, Boon P, Buyse G, et al. Steroids in intractable childhood epilepsy: clinical experience and review of the literature. Seizure. 2005;14(6):412-421.

32. Grosso S, Farnetani M, Mostardini R, Cordelli D, Berardi R, Balestri P. A comparative study of hydrocortisone versus deflazacort in drug-resistant epilepsy of childhood. Epilepsy Res. 2008;81(1):80-85.

33. Inui T, Kobayashi T, Kobayashi S, et al. Efficacy of long term weekly ACTH therapy for intractable epilepsy. Brain Dev. 2015;37(4):449-454.

34. Kalra V, Sharma S, Arya R. ACTH therapy in refractory generalized epilepsy. Indian J Pediatr. 2009;76(1):91-93.

35. Chatterjee A, Mundlamuri RC, Kenchaiah R, et al. Role of pulse methylprednisolone in epileptic encephalopathy: A retrospective observational analysis. Epilepsy Res. 2021;173:106611.

36. Charuvanij A, Ouvrier RA, Procopis PG, Antony JH, Fagan ER. ACTH treatment in intractable seizures of childhood. Brain Dev. 1992;14(2):102-106.

37. Bakker DP, Catsman-Berrevoets CE, Neuteboom RF. Effectiveness of a hybrid corticosteroid treatment regimen on refractory childhood seizures and a review of other corticosteroid treatments. Eur J Paediatr Neurol. 2015;19(5):553-560.

38. Nasiri J, Sarajan A, Salari M, Sedghi M. Therapeutic Effects of Adrenocorticotropic Hormone ACTH in Children with Severely Intractable Seizure. Iran J Child Neurol. 2017;11(3):19-26.

39. Gofshteyn JS, K KG, Marquis BO, et al. Measurable outcomes for pediatric epileptic encephalopathy: a single-center experience with corticosteroid therapy. Epileptic Disord. 2021;23(1):111-122.

40. Gobbi G, Loiacono G, Boni A, Marangio L, Verrotti A. Can ACTH therapy improve the long-term outcome of drug-resistant frontal lobe epilepsy? Epileptic Disord. 2014;16(2):185-190.

41. Pieribone VA, Tsai J, Soufflet C, et al. Clinical evaluation of ganaxolone in pediatric and adolescent patients with refractory epilepsy. *Epilepsia.* 2007;48(10):1870-1874.

42. Knight EMP, Amin S, Bahi-Buisson N, et al. Safety and efficacy of ganaxolone in patients with CDKL5 deficiency disorder: results from the double-blind phase of a randomised, placebo-controlled, phase 3 trial. *Lancet Neurol.* 2022;21(5):417-427.

43. Rosenthal ES, Claassen J, Wainwright MS, et al. Brexanolone as adjunctive therapy in super-refractory status epilepticus. *Ann Neurol.* 2017;82(3):342-352.
